# Banded mongooses avoid inbreeding when mating with members of the same natal group

**DOI:** 10.1111/mec.13253

**Published:** 2015-06-11

**Authors:** Jennifer L. Sanderson, Jinliang Wang, Emma I. K. Vitikainen, Michael A. Cant, Hazel J. Nichols

**Affiliations:** ^1^ College of Life and Environmental SciencesUniversity of ExeterPenryn CampusPenrynCornwallTR10 9FEUK; ^2^ Institute of ZoologyZoological Society of LondonRegent's ParkLondonNW1 4RYUK; ^3^ School of Natural Science and PsychologyLiverpool John Moores UniversityLiverpoolL3 3AFUK

**Keywords:** breeding systems, cooperative breeding, dispersal, inbreeding avoidance

## Abstract

Inbreeding and inbreeding avoidance are key factors in the evolution of animal societies, influencing dispersal and reproductive strategies which can affect relatedness structure and helping behaviours. In cooperative breeding systems, individuals typically avoid inbreeding through reproductive restraint and/or dispersing to breed outside their natal group. However, where groups contain multiple potential mates of varying relatedness, strategies of kin recognition and mate choice may be favoured. Here, we investigate male mate choice and female control of paternity in the banded mongoose (*Mungos mungo*), a cooperatively breeding mammal where both sexes are often philopatric and mating between relatives is known to occur. We find evidence suggestive of inbreeding depression in banded mongooses, indicating a benefit to avoiding breeding with relatives. Successfully breeding pairs were less related than expected under random mating, which appeared to be driven by both male choice and female control of paternity. Male banded mongooses actively guard females to gain access to mating opportunities, and this guarding behaviour is preferentially directed towards less closely related females. Guard–female relatedness did not affect the guard's probability of gaining reproductive success. However, where mate‐guards are unsuccessful, they lose paternity to males that are less related to the females than themselves. Together, our results suggest that both sexes of banded mongoose use kin discrimination to avoid inbreeding. Although this strategy appears to be rare among cooperative breeders, it may be more prominent in species where relatedness to potential mates is variable, and/or where opportunities for dispersal and mating outside of the group are limited.

## Introduction

**Figure 1:**
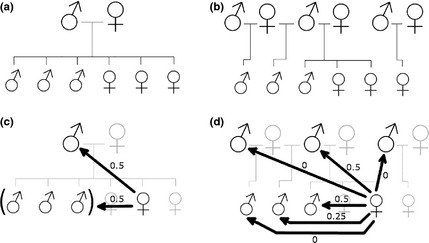
Differences in within‐group relatedness structure between meerkats (*Suricata suricatta*) and banded mongooses (*Mungos mungo*) may be attributable to differences in reproductive skew. Schematics of single breeding attempts within (a) meerkat and (b) banded mongoose social groups are shown with lines representing pedigree. Relatedness values of a single philopatric female to within‐group males after this single breeding attempt are shown for social groups of (c) meerkats and (d) banded mongooses. Meerkats have high reproductive skew with a stable breeding pair, while banded mongooses breed promiscuously with low reproductive skew; philopatric meerkat females do not have access to unrelated mating partners within their social group (except for immigrant males), whereas philopatric banded mongoose females do.

Breeding between relatives leads to inbreeding depression through an increase in offspring homozygosity and a decrease in fitness (Charlesworth & Charlesworth 1987; Frankham 1995; Keller & Waller 2002); hence, inbreeding avoidance is widespread (Pusey & Wolf 1996). The likelihood of encountering relatives as potential mates is particularly high in stable and/or isolated populations such as those of cooperative breeders which live in extended family groups. For cooperative breeders, within‐group relatedness is particularly high in groups where there is a single dominant breeding pair, as natal individuals are mostly first‐order relatives (e.g. meerkats; Fig. 1a,c). Here, inbreeding is most commonly avoided through sex‐biased philopatry: members of one sex disperse to breed elsewhere, while members of the other sex remain in their natal group, preferentially breeding with immigrants or members of neighbouring groups (e.g. meerkats: O'Riain *et al*. 2000; Young *et al*. 2007; pied babblers: Nelson‐Flower *et al*. 2012; purple‐crowned fairy‐wrens: Kingma *et al*. 2013; see reviews in Koenig & Haydock 2004; Lukas & Clutton‐brock 2011). However, in many species, groups contain multiple breeders of both sexes (Hodge 2009), and the degree of relatedness between natal individuals may range from very low (close to zero) to very high (0.5 or higher) (e.g. banded mongooses; Fig. 1b,d). These circumstances might favour the evolution of kin discrimination systems that allow individuals to reproduce within their natal group and yet avoid breeding with siblings or other close relatives.

The benefits of inbreeding avoidance will typically differ for male and female breeders because of sex differences in reproductive investment; in particular, the energetic and opportunity costs of producing poor‐quality offspring (Trivers 1972; Waser *et al*. 1986; Haig 1999). In mammals, the high costs of gestation and lactation for females mean that females could gain substantial benefits from inbreeding avoidance. Hence, females may be under particularly strong selection to evolve mechanisms that allow them to prevent fertilization by close male kin, for example by rejecting mating attempts or influencing the outcome of sperm competition (Hosken & Blanckenhorn 1998; Tregenza & Wedell 2002). Where male reproductive investment is low, male inbreeding avoidance may be expected only if mates are encountered simultaneously (Kokko & Ots 2006; Edward & Chapman 2011). However, in species where males invest heavily in courtship, mating or parental care, males may also experience substantial costs of inbreeding, and gain from channelling reproductive investment towards unrelated females even when encountered sequentially. It is important to note, however, that inbreeding is not always costly (Waser *et al*. 1986) or avoided (Olson *et al*. 2012) and individuals may in fact preferentially mate with relatives if it increases inclusive fitness (Puurtinen 2011; Szulkin *et al*. 2013). Although male mate choice has received growing attention in recent years (Lihoreau *et al*. 2008; Edward & Chapman 2011; Lemaître *et al*. 2012), little is known about the importance of, and possible interaction between, male and female mate choice strategies in inbreeding avoidance within social groups. Investigating this question requires the study of systems in which male mating effort and the level of female control over paternity can be readily observed and quantified.

**Figure 2:**
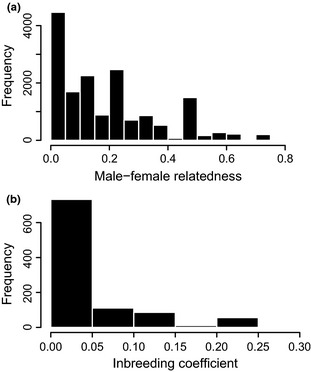
Histograms of (a) pairwise relatedness values from within‐group male–female pairs and (b) offspring inbreeding coefficients. (a) Estimates of pedigree‐based relatedness from adult (aged >1 year) males and females present within 419 observed breeding attempts (total number of possible pairs = 16 327; including 268 unique male identities and 185 unique female identities). (b) Pedigree‐based inbreeding coefficients from 1001 offspring with assigned parents. Note that one individual had an inbreeding coefficient of 0.375 but is excluded from the figure because it was not visible at this scale.

Here, we investigate male mate choice and female control of paternity as potential mechanisms of within‐group inbreeding avoidance in a wild population of banded mongooses (*Mungos mungo*). Banded mongooses are cooperative breeders that live in stable groups of 5–30 individuals in which both sexes often breed within their natal group and many remain as breeders within their natal group for their whole lives (Nichols *et al*. 2010; Cant *et al*. 2013). Within groups of banded mongooses, multiple (1–10) females enter oestrous synchronously, typically in the same week (Hodge *et al*. 2011). Females usually carry three foetuses per term (Cant 2000) and give birth synchronously (usually on the same day; Hodge *et al*. 2009) which creates large communal litters of up to 30 pups (Gilchrist 2006) which are then cared for communally by the whole group (Cant 2003). During group oestrus, each female is followed closely by one or more mate‐guards for periods of up to several days (Nichols *et al*. 2010). This mate‐guarding increases the chances of successful mating, but females often reject the mating attempts of mate‐guards and non‐mate‐guards still gain a share of paternity through sneak mating events with guarded females (Cant 2000; Nichols *et al*. 2010). Females have been observed to mate with multiple males (up to 5) in a single breeding attempt (Cant 2000) and are often guarded by different males in consecutive breeding attempts (Nichols *et al*. 2010). The consequence of these behaviours (and philopatry of both sexes) is substantial within‐group variation in pairwise relatedness between males and females of breeding age (Fig. 2).

When female banded mongooses do leave their natal group, they do so in single‐sex cohorts following forced evictions from older, more dominant females (Cant *et al*. 2001). Males also leave in single‐sex cohorts but can do so either voluntarily or following an eviction (Cant *et al*. 2013). A total of 13% and 12% of males and females have been observed to leave their natal group, respectively (Cant *et al*. 2013). New groups form when a cohort of dispersing males fuses with a cohort of females from a different natal group, either by taking over a new group and evicting all current males or (if both single‐sex cohorts have left their natal territory) by establishing a new territory. Migration between established groups is virtually absent with only three cases recorded in 18 groups over a period of 12 years (Cant *et al*. 2013). Although mating is skewed towards older individuals, both male and female banded mongooses are capable of breeding at 1 year of age (Cant 2000; Nichols *et al*. 2010) and do so often in the presence of their own parents. Females regularly conceive to close relatives including fathers and brothers (27% conceiving to a male related by 0.25 or more; Nichols *et al*. 2014). However, whether they do this less often than expected under random mating (as would be the case if males and/or females exercise inbreeding avoidance) remains unclear. In this study, we use a combination of behavioural and genetic data to investigate patterns of male mate choice and female control of paternity to determine whether banded mongooses exercise any inbreeding avoidance strategies. Specifically, we address four questions: (1) Is there evidence of costs associated with inbreeding in banded mongooses? (2) Is there evidence of inbreeding avoidance in banded mongooses? (3) Is there evidence that males avoid inbreeding by directing mating effort towards unrelated females? (4) Is there evidence that females avoid inbreeding through rejecting related mating partners?

## Materials and methods

### Study site and data collection

Behavioural and genetic data were collected from wild mongooses inhabiting the Mweya Peninsula, Queen Elizabeth National Park, Uganda (0°12′S, 27°54′E) between May 1997 and September 2013. Details of vegetation and climate are available elsewhere (Cant *et al*. 2013). All individuals in the population were habituated to the presence of human observers at 2–4 m, allowing the collection of detailed behavioural data without any measureable effect of observer presence. Groups were visited every 2–4 days to collect behavioural and life history data. Accurate ages (±2 days) were known for the majority (90%) of the population. Where accurate ages were not known (e.g. for immigrants or new groups), individuals were simply classified as pups, juveniles or adults according to their size, body mass and/or tooth wear (note that the majority of analyses were limited to adults) (Cant 2000). This research was carried out under licence from the Uganda National Council for Science and Technology, and all procedures were approved by the Uganda Wildlife Authority.

One or two individuals within each group were fitted with a radio collar weighing 27 g (Sirtrack Ltd., New Zealand) with a 20‐cm whip antenna (Biotrack Ltd., UK). All individuals within the population were marked, either with a unique shave pattern on their back or with a colour‐coded plastic collar. Young individuals (aged <6 months) were marked using commercially available blonde hair dye (L'Oreal, UK) to create a unique pattern on their backs. Pups were trapped within 2 weeks of emerging from the den (aged 30–50 days), and all individuals within the population were trapped every 3–6 months to maintain collars and shave/hair‐dye patterns. Individuals were trapped using box traps (67 × 23 × 23 cm; Tomahawk Live Trap Co., Tomahawk, WI, USA) and anaesthetized using isoflurane applied through a silicon face mask or (for individuals <6 months old) using intramuscular injections of 1 mg/kg of ketamine and 0.8 mg/kg of medetomidine, followed by an injection of 0.8 mg/kg of atipamezole after handling (further details of trapping protocol are given elsewhere; ketamine: Hodge 2007; isoflurane: Jordan *et al*. 2010).

On first capture, permanent identification was made possible using either a uniquely coded tattoo or a pit tag (TAG‐P‐122IJ, Wyre Micro Design Ltd., UK). A 2‐mm skin sample for genetic analysis was collected from the end of the tail using sterile surgical scissors. This process caused little or no bleeding. After sample collection, the end of the tail was treated with a dilute solution of potassium permanganate to reduce the chances of infection. This trapping protocol was used over 8000 times during the course of study, and genetic samples were collected from 1786 individuals without any adverse effects.

#### Observations of mating behaviour

Groups were visited daily during 211 group oestrus periods between April 2003 and September 2013 for observations of mating behaviour. The ‘group oestrous’ period (i.e. the time from the first to the last day on which mating and mate‐guarding was observed in a particular breeding attempt) lasted 3.1 ± 0.1 days (mean ± SE, from 211 oestrous periods). During group oestrus, each oestrous female is closely followed and guarded by a single male ‘*mate‐guard*’ for periods that last from several hours to several consecutive days. Mate‐guards defend their associated female from attempts to mate by other males by snapping, lunging and pouncing towards approaching males (Nichols *et al*. 2010). These mate‐guarding behaviours are conspicuous and are easy to identify (Cant 2000). During each observation session (1–5 h; 1–2 sessions per day), all males in the group were classified as mate‐guards or nonmating males (Cant 2000; Nichols *et al*. 2010) based on whether or not they engaged in mate‐guarding behaviours during the observation session. For mate‐guarding males, the identity of their guarded female was also recorded.

### Genetic analysis

DNA was extracted from tail tips by lysis with proteinase K, followed by phenol–chloroform purification (Sambrook *et al*. 1989) or using DNA extraction kits (Qiagen^®^ Tissue and Blood Kit). Samples were genotyped at up to 43 microsatellite loci, isolated from a variety of carnivore species, including the banded mongoose. Genotyping was conducted following Nichols *et al*. (2010) or (post‐2010) using multiplex PCRs (Qiagen^®^ Multiplex PCR Kit, UK) with fluorescent‐labelled forward primers and was visualized through fragment size analysis on an ABI 3730 DNA Analyzer. PCR conditions followed the Qiagen^®^ Multiplex PCR Kit recommendations (but were conducted in 12‐μL reactions), with an annealing temperature of 57°C. Full details of the 43 microsatellites used in this study alongside primer sequences, multiplex sets and PCR conditions are given in the Appendix S1.1 (Supporting information).

Deviations from Hardy–Weinberg equilibrium (HWE) and linkage disequilibrium (LD) were tested using Genepop 4.3 (Raymond & Rousset 1995; Rousset 2008). When tests were carried out on the full data set 33/43 loci and 826/903 pairs of loci were found to deviate from HWE and LD, respectively (see Appendix S1.1, Supporting information: Table S1.1.4). However, when tests were carried out on 300 randomized subpopulations of nonrelatives, no loci or pairs of loci were found to consistently deviate from HWE or LD (see Appendix S1.1, Supporting information: Tables S1.1.4 and S1.1.5). All loci were manually checked for sex linkage by comparing a subset of male and female genotypes. Full details of allele frequencies as well as expected and observed heterozygosity values are given in the Appendix S2 (Supporting information).

We generated a 9‐generation‐deep pedigree using familial relationships within the banded mongoose research project study population inferred using field observations, individual genotypes and two freely available programs: masterbayes 2.51 (Hadfield *et al*. 2006), which was implemented in r 3.1.1 (R Core Team 2013), and colony 2.0.5.7 (Jones & Wang 2010). Full details of pedigree construction are given in the Appendix S1.2 (Supporting information).

In brief, we first used masterbayes (Hadfield *et al*. 2006) to assign parents to 2633 individuals classified as offspring (i.e. individuals that were observed being born into the population, 2633 from a total of 2878 individual recorded in the population), of which 1593 were genotyped. All females (aged >6 months) present in the offspring's natal group at birth were included as candidate mothers, and all males (aged >6 months) present in the study population at conception were included as candidate fathers to allow for extra‐group mating. We also included the following phenotypic predictors of parentage: whether or not a female was recorded as giving birth, if a male was in the offspring's natal group prior to birth, and the age and quadratic age of both males and females. The numbers of unsampled candidate mothers and fathers were estimated in the parentage assignment model. Genotyping error rates were calculated manually from samples that were genotyped in duplicate following Hoffman & Amos (2005). Allele frequencies were calculated in cervus 3.0.7 (Kalinowski *et al*. 2007) using the full genotype data set. These genotyping error rates and allele frequencies were provided in the model specification. The Markov chain Monte Carlo estimation chain was run for 1 500 000 iterations with a thinning interval of 500 and a burn‐in of 500 000. No further prior distributions were specified, and default improper priors were used. Successive samples from the posterior distribution had low autocorrelation (*r* < 0.01).

Second, sibships were constructed in colony (Jones & Wang 2010) by partitioning all 1787 genotyped individuals (including offspring, founders and immigrants) into full‐ and half‐sibship groups with or without parentage assignments, using a maximum‐likelihood method. The same candidate parent criteria were used as above to generate candidate father list, candidate mother list, paternal exclusion list and maternal exclusion list as input into colony. No maternal or paternal sibships were excluded. A weak sibship prior of 1.5 for both maternal and paternal average sibship size was included to limit false‐positive sibship assignments, and the probabilities that the true mother and father were in the candidate lists were both set as 0.8 (see Appendix S1.2, Supporting information: Fig. S1.2.1).

Parentage assignment was accepted with ≥0.8 probability in both masterbayes and colony. masterbayes parentage assignments were accepted first (1474 assigned maternities and 1397 assigned maternities, note that no ungenotyped individuals were confidently assigned parentage), and colony parentage assignments were then added where masterbayes had failed to assign parentage (a further 29 maternities and 45 paternities). Note that of the 1200 and 1029 cases in which both masterbayes and colony assigned maternity and paternity, only 55 and 69 were mismatched, respectively. Following this, we used the full‐sibships assigned using colony to infer maternity and paternity to a further 67 and 34 offspring, respectively (see Appendix S1.2, Supporting information for further details). These assignment rules allowed us to infer a 9‐generation‐deep pedigree, which includes 1570 maternities and 1476 paternities.

Using the same panel of genetic markers for parentage assignment and for calculating levels of relatedness has been shown to bias paternity assignments towards unrelated fathers in some cases (Wang 2010). We minimized the probability of encountering such biases using a large panel of markers for parentage analysis (43 microsatellites) which allowed for high confidence of parentage assignment in almost all cases; of the 1083 offspring genotyped during the period of behavioural observations (between April 2003 and September 2013), 986 and 955 (91% and 88%) were assigned paternity at ≥0.8 and ≥0.95, respectively (see Appendix S1.3, Supporting information for further details of testing for biases in parentage assignment). Furthermore, where possible, we verified our genetic data using behavioural observations of mate‐guarding patterns, which are not subject to such biases.

### Statistical analyses

#### Is there evidence of costs associated with inbreeding in banded mongooses?

To test for possible costs associated with inbreeding in banded mongooses, we modelled its effect on two variables that are likely to be associated with fitness: yearling body mass and survival to 1 year. Pedigree‐based inbreeding coefficients (*F*) were available for 1001 individuals (with assigned parents) born between March 2003 and September 2013. In total, 425 of the individuals included in these analyses had nonzero inbreeding coefficients.

Overall, 777 observations of body mass were available from 210 yearlings (aged between 350 and 370 days) from 79 breeding attempts and nine social groups. This yearling body mass was fitted as a response in a generalized linear mixed model (GLMM) with inbreeding coefficient as the main predictor of interest along with age in days to control for differences in age at measurement. Further to this, data on survival to 1 year of age were available for 839 individuals from 183 breeding attempts in 13 social groups. This survival to independence was fitted as a binomial response in a GLMM, again with inbreeding coefficient as the main predictor of interest. Mean daily rainfall in the 30 days prior to birth, maternal age (months), the number of pups born in the same litter as the observed individual, and group size at the time of birth (number of individuals aged >1 year) were also fitted as fixed effects in both models to control for their possible effects on both response traits. Social group, breeding attempt, maternal identity and paternal identity were fitted as random factors in both models to control for repeated measures as well as an individual identity in the body mass model to control for repeated observations of the same individual.

#### Is there evidence of inbreeding avoidance in banded mongooses?

To test whether banded mongooses preferentially mate with nonrelatives from within their social group, we compared pairwise relatedness estimates from observed breeding pairs with pairwise relatedness estimates from simulated male–female dyads under random mating. Specifically, we created randomizations of male–female dyads by assigning each female (with assigned maternity) to a random adult male (aged >1 year) from within the same social group. If a female had multiple pups sired by the same male within a breeding attempt, then this was counted as a single male–female breeding pair and the female was only assigned one random male within each permutation. If a female had pups assigned to more than one male within a breeding attempt, she was assigned the same number of random males. Data were available from 624 successful breeding pairs of banded mongooses from 196 breeding attempts in 16 different social groups. However, we limited this data set to 269 breeding pairs which satisfied the following criteria: (1) mother had both parents confidently assigned (452/624 observations); (2) at least 80% of candidate fathers had confidently assigned parents (395/624 observations); (3) the male with assigned paternity was from the same group as the female assigned maternity (i.e. within‐group mating; 400/624 observations). Exclusion criteria 1 and 2 reduced noise associated with including pedigree‐derived relatedness coefficients from individuals with unknown parentage in randomizations while exclusion criteria 3 allowed us to test for inbreeding avoidance in the absence of any effects of extra‐group mating. Within each permutation, we calculated the mean pairwise relatedness of 269 randomized male–female dyads. Raw values from the 269 observed male–female dyads were compared to null distributions generated from 10 000 permutations of the data to derive a one‐tailed *P*‐value.

As we are interested in inbreeding avoidance in the absence of any cues of familiarity (i.e. within‐ vs. extra‐group individuals and/or natal‐ vs. non‐natal individuals), we repeated these simulations limiting the data set to 137 breeding attempts where both all adult males and all adult females were observed to have been born within the same social group. This further conservative analysis allowed us to clarify whether inbreeding avoidance occurs in the absence of cues of familiarity which may be present in newly formed groups or those which have recently accepted immigrants. Here, estimates of relatedness were available from 439 observed male–female dyads which were then limited to 201 dyads following the same criteria as above (criteria 1: 328/439; criteria 2: 306/439; criteria 3: 276/439); raw values from these 201 observed male–female dyads were compared to null distributions generated from 10 000 permutations of this data set of natal individuals to derive a one‐tailed *P*‐value.

#### Is there evidence that males avoid inbreeding by directing mating effort towards unrelated females?

To test whether male banded mongooses preferentially direct guarding effort towards unrelated females, we compared pairwise relatedness estimates from observed guard–female dyads with pairwise relatedness estimates from simulated guard–female dyads under random mating. Specifically, we created randomizations of guard–female dyads by assigning males that had been observed mate‐guarding to a random guarded female from within the same oestrus event. If a male was observed to guard more than one female within an oestrus event, he was randomly assigned the same number of females; similarly, if a female was guarded by more than one male, then the same number of guards was assigned to her. Data were available from 1074 observed guard–female pairs from 212 oestrus events in 13 different social groups. However, (similar to the analyses above) we limited this data set to 649 guard–female pairs which satisfied the following criteria: (1) the mate‐guard had confidently assigned parents (866/1074 observations), and (2) at least 80% of candidate females had confidently assigned parents (738/1074 observations). Within each permutation, we calculated the mean pairwise relatedness of 684 randomized guard–female dyads. Raw values were compared to null distributions generated from 10 000 permutations of the data to derive a one‐tailed *P*‐value.

Again, as we are interested in whether or not male banded mongooses are able to direct their mating effort towards unrelated females in the absence of simple cues of familiarity (i.e. group membership), we repeated these simulations limiting the data set to 175 breeding attempts where all adult females were observed to be born within the same natal group. Here, estimates of relatedness were available from 842 observed guard–female dyads which were then limited to 481 dyads following the same criteria as above (criteria 1: 686/842; criteria 2: 548/842); raw values from these 481 observed guard–female dyads were compared to null distributions generated from 10 000 permutations of this data set of natal individuals to derive a one‐tailed *P*‐value.

#### Is there evidence that females avoid inbreeding through rejecting related mating partners?

Previous behavioural observations indicate that females sometimes reject the copulation attempts of their mate‐guards (Cant 2000) and so may plausibly influence control over the distribution of paternity among males by rejecting mating attempts. Females could also exercise cryptic mate choice by influencing the probability of fertilization or successful implantation postcopulation. To evaluate the degree to which females can influence the distribution of paternity, we investigated (i) whether males guarding unrelated females were more likely to be successful in gaining paternity than males guarding related females and (ii) where mate‐guards were not successful in gaining paternity, we compared the relatedness of the mate‐guard and extra‐pair paternity male (EPP) to the female to test whether females were ‘upgrading’ to males they were less related to.

In total, 234 mate‐guard identities were observed for 171 females which were confidently assigned at least one offspring within the 40–80 days following observed oestrus (note that females were often guarded by more than one male per oestrus period). Within each of these mate‐guard–female pairs, the mate‐guard was categorized as ‘successful’ or ‘unsuccessful’ at reproducing with that female if it did or did not gain paternity, respectively. We further limited this data set to 159 pairs of mate‐guard and female identities which both had confidently assigned parents and were of known age/age rank (mate‐guard with assigned parents: 193/234; female with assigned parents: 187/234; guard known age rank: 212/234; female known age: 220/234). These exclusion criteria reduced noise associated with using pedigree‐derived relatedness from individual without assigned parents and allowed us to test for variation in mate‐guard success while controlling for any effects of age (Nichols *et al*. 2010). If females do exert control over paternity as a strategy to avoid inbreeding, then we expect males to be more successful when guarding an unrelated female. Paternity success was fitted as a binomial response in a GLMM with guard–female relatedness as the main predictor of interest. Male age rank, female age, sex ratio and the number of days spent guarding were also fitted as fixed effects to control for any effects on mate‐guard success. To exclude any possibility that females may use natal group membership as cues to relatedness when exerting control over paternity of their offspring, we repeated this analysis limited to 116 mate‐guard–female pairs in which all within‐group males were observed to be born within the same natal group and the above criteria were again satisfied.

From the 234 observed guard–female pairs, 160 were of mate‐guard identities which did not match any offspring assigned to that female within that breeding attempt (i.e. indicative of extra‐pair paternity; EPP). This data set was limited to 114 mate‐guard–female pairs where the identities of parents were confidently assigned for the mate‐guard, female and the EPP male (mate‐guard with assigned parents: 138/160; female with assigned parents: 131/160; EPP male with assigned parents: 138/160). Furthermore, we excluded another 12 cases where there were 2 assigned EPP identities which did not match the mate‐guard identity to allow for a direct pairwise comparison per breeding event (leaving a total of 102 paired relatedness values for analysis). We compared the relatedness of mate‐guard–female pairs with that of EPP male–female pairs using paired *t*‐tests. Females may avoid inbreeding either by mating with unrelated males within their own group or by mating with extra‐group males (Nichols *et al*. in press). To examine whether females exert control over paternity towards unrelated males while still mating within their own group, we categorized the EPP males as within‐group (WG) or extra‐group (EG) and carried out 2 further *t*‐tests limited to either within‐group or extra‐group EPP males. We also repeated these analyses with data limited to 89 guard–female pairs in social groups where all males were known to be from the same natal group and the above criteria were satisfied.

All statistical analyses were carried out using r 3.0.1 (R Core Team 2013). We used GLMMs to control for repeated measures within social groups, breeding attempts and individuals fitted using the lme4 package (Bates *et al*. 2013). Binomially distributed response variables were analysed with a logit link function. Explanatory variables were sequentially dropped from the model until only those variables explaining significant variation (*P* < 0.05) remained following Crawley (2012). All dropped variables were then individually put back into the minimal model to determine their level of nonsignificance. Social group, breeding attempt and male and female identities were included as random effects in all analyses where appropriate.

## Results

### Is there evidence of costs associated with inbreeding in banded mongooses?

**Figure 3:**
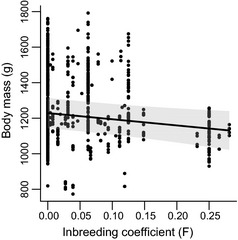
Relationship between inbreeding and body mass (g) in banded mongooses aged between 350 and 370 days. Dots show raw values. Line and shaded area show predicted mean and standard error estimated from a GLMM controlling for a significant effect of age.

Yearling body mass decreased with increase in the inbreeding coefficient (GLMM; $\chi_{\left(1\right)}^{2}$ = 5.29, *P* = 0.021; Fig. 3) suggestive of a cost to inbreeding. Variation in age at capture had an effect on weight (GLMM; $\chi_{\left(1\right)}^{2}$ = 11.64, *P* = 0.0006), but there was no effect of the number of pups, rainfall, group size or maternal age on pup body mass at 1 year of age (Table 1).

**Table 1:** Effects of inbreeding on body mass and survival to 1 year of age. Significant results are given in bold. Social group, litter, paternal and maternal identities were included as random effects in both models as well as individual identity in the model testing yearling body mass

Explanatory terms	Yearling body mass (aged 350–370 days)			Survival to 1 year of age		
	Effect size ± SE	$\chi^{2}$	*P*	Effect size ± SE	$\chi^{2}$	*P*
Inbreeding coefficient	**−347.9 ± 143.4**	**5.29**	**0.02**	−0.03 ± 0.23	<0.001	0.99
Maternal age	0.70 ± 0.42	2.84	0.09	0.00 ± 0.00	0.46	0.50
Group size	2.23 ± 2.08	1.11	0.29	−0.01 ± 0.02	0.52	0.47
Number of pups	1.13 ± 2.30	0.22	0.64	0.02 ± 0.01	1.51	0.22
Rainfall	−4.51 ± 6.62	0.64	0.42	**0.20 ± 0.07**	**8.09**	**0.004**
Age (days)	**1.15** ± **0.22**	**11.64**	**<0.001**	NA		
Constant	807.5 ± 127.9			−1.31 ± 0.22		

We found no effect of inbreeding on the likelihood of survival to 1 year of age (GLMM; $\chi_{\left(1\right)}^{2}$ < 0.001, *P* = 0.99), nor was there any effect of group size, maternal age or the number of pups (Table 1). Banded mongooses were more likely to survive to 1 year of age when daily rainfall 30 days prior to their birth was high (GLMM; $\chi_{\left(1\right)}^{2}$ = 8.09, *P* = 0.004).

### Is there evidence of inbreeding avoidance in banded mongooses?

**Figure 4:**
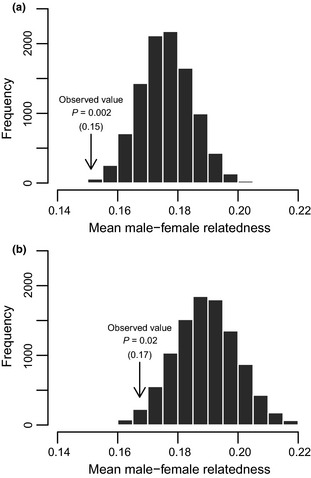
Randomization histograms of the null distribution of mean male–female pairwise relatedness if females were to randomly mate with adult males within their group; (a) when all breeding attempts are considered and (b) when only breeding attempts with single‐sex cohorts from the same natal group were considered.

If male and/or female banded mongooses use kin discrimination to avoid mating with relatives and the associated inbreeding costs, we expect females to mate with males that are less related to them than expected under random pairing. The observed mean relatedness between breeding male–female pairs was lower than expected by chance both when all data were considered (observed value = 0.15, null distribution mean = 0.18, *P* = 0.002; Fig. 4a) and when data were limited to breeding attempts where all adult males and all adult females were from the same natal group (observed value = 0.17, null distribution mean = 0.19, *P* = 0.019; Fig. 4b).

### Is there evidence that males avoid inbreeding by directing mating effort towards unrelated females?

**Figure 5:**
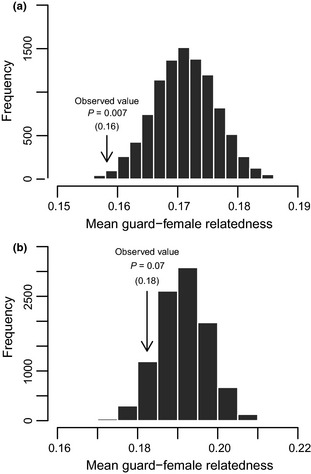
Randomization histograms of the null distributions of mean guard–female pairwise relatedness if males were to randomly guard receptive females within their group; (a) when all breeding attempts were considered and (b) when analyses were restricted to breeding attempts where all females were from that same natal group.

If males direct mating effort towards unrelated females, we predict males to guard females that are less related to them than expected under random pairing. The observed mean relatedness between mate‐guards and guarded females was lower than expected by chance when analysing the complete data set (observed value = 0.16, null distribution mean = 0.17, *P* = 0.007; Fig. 5a). However, when analysis was limited to breeding attempts where all females were from the same natal group (i.e. mate‐guards had no access to simple cues of familiarity), we only found a trend for males to mate‐guard females that are less related to them than expected by chance (observed value = 0.18, null distribution mean = 0.19, *P* = 0.072; Fig. 5b).

### Is there evidence that females avoid inbreeding through rejecting related mating partners?

Mate‐guards were no more likely to be successful at gaining paternity when guarding a female of lower relatedness (GLMM; $\chi_{\left(1\right)}^{2}$ = 3.01, *P* = 0.083), implying that females do not exert control over paternity of their offspring with respect to relatedness (through either pre‐ or postcopulatory mate choice). Older ranked guards were more likely to be successful at gaining paternity than younger age‐ranked guards (GLMM; $\chi_{\left(1\right)}^{2}$ = 6.35, *P* = 0.012), and increased number of days spent guarding increased a guards' chance of success ($\chi_{\left(1\right)}^{2}$ = 6.51, *P* = 0.011). Neither female age nor sex ratio had an effect on a mate‐guard's likelihood of gaining reproductive success with the guarded female (Table 2). When analyses were restricted to females that had no access to simple rules of familiarity (all within‐group males were of the same natal group), we obtained qualitatively similar results (Table 2).

**Table 2:** Factors affecting mate‐guard likelihood of gaining paternity with guarded female for (i) all females and (ii) only females with no access to simple rules of familiarity (i.e. relatedness dependent on natal group membership). Effect sizes are given on the logit scale. Significant results are given in bold. Social group, breeding attempt, guard and female identities were included as random effects in both models

Explanatory terms	All females			Females with no access to familiarity cues of relatedness		
	Effect size ± SE	$\chi^{2}$	*P*	Effect size ± SE	$\chi^{2}$	*P*
Guard–female relatedness	−2.60 ± 1.63	3.01	0.083	0.73 ± 1.48	0.24	0.63
Male age rank	**−0.20 ± 0.09**	**6.35**	**0.012**	−0.14 ± 0.09	2.88	0.089
Female age	0.02 ± 0.01	1.81	0.18	0.02 ± 0.01	3.63	0.057
Group sex ratio (% male)	3.89 ± 3.19	1.67	0.20	4.31 ± 4.41	1.07	0.30
Number of guarding days	**0.52 ± 0.22**	**6.51**	**0.011**	**0.81 ± 0.29**	**9.86**	**0.0017**
Constant	−1.05 ± 0.58			−2.76 ± 0.62		

**Figure 6:**
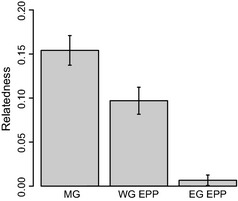
Relatedness estimates of a female to the observed mate‐guard (MG;* n* = 102), within‐group extra‐pair paternity (WG EPP;* n* = 81), and extra‐group extra‐pair paternity (EG EPP;* n* = 21) where the paternal identity did not match the observed mate‐guard identity. Bars show mean values and error bars show standard errors. Female relatedness to the EPP male was significantly lower than that to the observed mate‐guard with both within‐ and extra‐group matings.

When paternity was assigned to a male which did not match the observed mate‐guarding male (i.e. extra‐pair paternity; EPP), females were less related to the EPP male than they were to their mate‐guard (*t*‐test: *t*_101_ = 4.19, *P* < 0.001; Fig. 6). Furthermore, this difference remained significant when considering only within‐ or extra‐group EPPs (t‐test; within‐group extra‐pair paternity: *t*_80_ = 2.47, *P* = 0.016; extra‐group extra‐pair paternity: *t*_20_ = 4.54, *P* < 0.001; Fig. 6). Again, qualitatively very similar results were obtained when these analyses were restricted to females that had no simple familiarity cues to relatedness (*t*‐tests: mate‐guard vs. extra‐pair paternity: *t*_88_ = 4.03, *P* < 0.001; mate‐guard vs. within‐group extra‐pair paternity: *t*_71_ = 2.60, *P* = 0.011; mate‐guard vs. extra‐group extra‐pair paternity: *t*_16_ = 3.85, *P* = 0.001).

## Discussion

Our findings demonstrate patterns of inbreeding avoidance in a wild population of banded mongooses. To our knowledge, we are the first to describe a cooperative breeding system where inbreeding avoidance can occur even in the absence of dispersal or mating between groups. We found that inbred pups were lighter at 1 year of age. Given that early‐life body mass is a strong predictor of adult fecundity (Hodge 2005), this is highly indicative of a cost to inbreeding in banded mongooses. Successfully breeding pairs, identified through genetic parentage analysis, were found to be less related than expected under random mating. Male banded mongooses directed mating effort (mate‐guarding) towards unrelated females, indicating that males are able to discriminate between relatives and use selective mate choice to avoid inbreeding. Males guarding unrelated females were no more likely to be successful than males guarding related females. However, when mate‐guards were unsuccessful, we found that paternity was assigned to males that were less related to the female than her mate‐guard. These results suggest that although males preferentially direct their mating effort towards unrelated females, females themselves may also actively avoid inbreeding through exerting control over paternity. Together, our results are strongly suggestive of an ability to discriminate between relatives and avoid inbreeding for both male and female banded mongooses even when mating with individuals from the same natal group.

One potential difficulty for studies of inbreeding is that it may be more difficult to assign paternity of offspring to males that are more closely related to their female mates, leading to inflated estimates of the relative reproductive success of unrelated compared to related males (Wang 2010). This may be particularly likely when the true father has not been sampled, resulting in an assignment being made at low confidence to the incorrect male. In the current study, 93% of candidate fathers were genotyped and 91% of offspring were confidently assigned paternity. Although we found a significant negative effect of parent relatedness on the confidence of masterbayes paternity assignment, the effect size was very small with parents that were first‐order relatives (i.e. *r* = 0.5) expected to have a paternity assignment with confidence reduced by 0.04 compared to paternity assignment between nonrelatives (i.e. *r* = 0) (see Appendix S1.3, Supporting information for further details). We interpret this as suggestive that any bias in paternity assignment towards unrelated males is unlikely to affect our downstream analyses given the high proportion of offspring assigned confident parentage in our pedigree. A second difficulty for inbreeding studies is that intense inbreeding depression, such as selective abortion and/or increased mortality of inbred pups, could generate results compatible with reproductive skew towards unrelated males if the highly inbred offspring of related males rarely survive. As female banded mongooses give birth synchronously in inaccessible underground dens, sampling or even counting offspring within the communal litter is impossible until they emerge at ~30 days of age (Cant *et al*. 2013). Therefore, we cannot reject the possibility that the results presented for questions 2 and 4 could also arise from differential survival between inbred and outbred pups. Unrelated mating pairs experiencing higher reproductive success could therefore reflect inbreeding avoidance, inbreeding depression or combination of the two. However, as the methods used to address question 3 only use behavioural data, there is still evidence for within‐group inbreeding avoidance even if differential survival accounts for the results presented for questions 2 and 4.

An individual's ability to choose an unrelated mating partner is reliant on accurate mechanisms of kin discrimination. This may be through rules of familiarity (Clarke & Faulkes 1999; Frommen *et al*. 2007) or self‐referential cues (Mateo 2010; Thünken *et al*. 2013). Where there are high levels of promiscuity and reproductive synchrony, such as in the banded mongoose (Cant 2000; Hodge *et al*. 2011), familiarity may be an unreliable indicator of relatedness and so individuals are more likely to use self‐referent cues to find an unrelated mating partner. Examples include major urinary proteins (MUPs, Hurst *et al*. 2001; Sherborne *et al*. 2007) and other odours linked to the major histocompatibility complex (MHC; Gerlach & Lysiak 2006; Havlicek & Roberts 2009; Leclaire *et al*. 2014). Banded mongooses use scent from anal gland secretions to communicate both within and between groups (Müller & Manser 2007; Jordan *et al*. 2010, 2011) and show marked differences between individual variation in scent profiles (Jordan *et al*. 2011), suggesting that they may use scent as a cue to relatedness (as seen in meerkats; Leclaire *et al*. 2012). Furthermore, banded mongooses emit highly frequent vocal contact calls which contain individually identifiable signatures (Jansen *et al*. 2013), and it is also possible that vocal signatures act as a cue to relatedness (Penn & Frommen 2010).

The costs of inbreeding are expected to be highest for individuals with high reproductive investment. For many species, the energetic costs associated with gamete production and offspring care mean that reproductive investment is highest in females (Trivers 1972; Haig 1999). However, males can also sometimes invest heavily in reproduction, through both mating effort and investment in offspring care. Male banded mongooses guard females for multiple consecutive days in order to gain access to paternity. This guarding behaviour involves costly aggressive interactions (Cant 2000; Nichols *et al*. 2010) and reduces the time available for foraging (Sanderson, pers. obs.). Furthermore, male banded mongooses also invest heavily in offspring care, often even more so than females (Hodge 2007). This high reproductive investment suggests that male banded mongooses may also experience high fitness costs associated with inbreeding, which could explain why males are observed to preferentially guard unrelated females. Male mate choice is also predicted to occur where there is variation in female quality and where receptive females are encountered simultaneously (Edward & Chapman 2011). Indeed, high levels of promiscuity within banded mongoose societies mean that males have access to females which vary in genetic compatibility, and the high degree of female reproductive synchrony seen within banded mongoose groups (Hodge *et al*. 2011) means that males do encounter receptive females simultaneously. The extent to which females synchronize breeding within groups could in fact promote male choice even in the absence of high male reproductive investment as male mating opportunities are limited by the fact that they can only guard one female at a time. Together, these factors are indicative of a breeding system where male choosiness is predicted and highlight the possibility that the nonrandom pairing seen in this study may be a result of male mate choice to avoid fitness costs associated with inbreeding.

The probability of reproductive success for guarding males (measured as whether or not a mate‐guard was assigned paternity) was found to be independent of relatedness to the guarded females, suggesting that females are no more likely to reject the mating attempts of related guards. However, where mate‐guards were unsuccessful, they lost paternity to males that were less related to the females than themselves. Although this pattern may be driven by differential offspring survival (see above), it indicates that females may direct paternity away from their mate‐guards when there is an opportunity to upgrade to a less related male. Where females are able to influence paternity of their offspring (e.g. through postcopulatory mechanisms such as sperm competition; Simmons 2005 and/or selective abortion; Thomas *et al*. 1985), this may also influence the optimal mate choice strategies of males (Tennenhouse 2014); males have little to gain through investment in mate‐guarding or fighting to monopolize access to a particular female if she then rejects him as a mate or reduces his fertilization success postcopulation. This means that males may be observed to preferentially direct mating effort towards unrelated females even in the absence of any inbreeding costs to themselves. However, given the high reproductive investment of male banded mongooses (both mate‐guarding and offspring care; Gilchrist & Russell 2007; Hodge 2007; Nichols *et al*. 2010), it seems more likely that male mate choice has evolved as a male inbreeding avoidance strategy rather than a response to female choice.

Individuals living within stable social groups frequently encounter close relatives as potential mates. How individuals respond to this can have profound effects on population processes. Previous studies of inbreeding avoidance in cooperatively breeding species have focused on reproductive suppression and sex‐biased philopatry (Blouin & Blouin 1988; Lukas & Clutton‐brock 2011; Nelson‐Flower *et al*. 2012). Although banded mongooses do sometimes breed with close relatives and often breed with more distant relatives (Nichols *et al*. 2014), we have shown here that individuals may also avoid inbreeding through selective mate choice. Banded mongooses do not exhibit sex‐biased philopatry; both sexes commonly breed within their natal group and remain there for their whole lives (Cant *et al*. 2013). Thus, the ability to discriminate between kin and nonkin within individuals of the same natal group may allow banded mongooses to avoid the potentially high costs of dispersal while still avoiding any fitness consequences of inbreeding. This mechanism of inbreeding avoidance is previously unknown in cooperative breeders (Lukas & Clutton‐brock 2011), but may be more important in species where there is variation in within‐group relatedness and where dispersal or extra‐group mating opportunities are limited.

## Funding

This work was funded by a grant from the Natural Environment Research Council (grant number NE/J010278/1).

J.L.S. and H.N. designed the research; H.N. carried out the genetic analyses; J.L.S. analysed the data with assistance from H.N.; J.W. carried out the sibship and parentage assignments in colony; J.L.S., M.A.C. and H.N. wrote the manuscript with comments from E.V. and J.W.; field data collection was carried out by E.V., M.A.C. and J.L.S.

## Data accessibility

DNA sequences for the microsatellite loci: GenBank Accession nos AF271115, AF271117, AF271118, AF271120, AY142693, AY142694, AY142696, AY142697, AY142700, AY142703, AY155580, AY090498, EU045417, EU045419, KP895833, KP895834, KP895835, KP895836, KP895837, KP895838, KP895839, KP895840, KP895841, KP895842, KP895843, KP895844, KP895845, KP895846, KP895848, KP895849, KP895850, KP895851, KP895852. JF746985, ERP000497, JF746989. See Table Appendix S1.1.1.1. (Supporting information) for further details.

Microsatellite genotypes, pedigree, relatedness estimates, inbreeding coefficients and behavioural data: Dryad doi:10.5061/dryad.gc371.

## Supporting information


**Appendix S1** (S1.1) Microsatellite information, (S1.2) construction of the Banded Mongoose Research Project pedigree, and (S1.3) testing for signs of bias in MasterBayes paternity assignment.

**Appendix S2** Observed and expected heterozygosities, allele frequencies and Fis from Genepop 4.3.
