# Supplementary material for: Banded mongooses avoid inbreeding when mating with members of the same natal group
Source: Mol Ecol. 2015 Jun 11;24(14):3738–51. doi: 10.1111/mec.13253 (PMC5008155; doi:10.1111/mec.13253)
Supplement: Supplementary file 1 — Appendix S1 (S1.1) Microsatellite information, (S1.2) construction of the Banded Mongoose Research Project pedigree, and (S1.3) testing for signs of bias in MasterBayes paternity assignment. [file MEC-24-3738-s001.docx]

**Supporting Information 1: Microsatellite information, construction of the Banded Mongoose Research Project pedigree, and testing for signs of bias in MasterBayes paternity assignment**

Jennifer L Sanderson, Jinliang Wang, Emma I K Vitikainen, Michael A Cant, & Hazel J Nichols

**Supporting information S1.1 Microsatellite information and tests for Hardy-Weinburg equilibrium and linkage disequilibrium**

**Supporting Information S1.2: Construction of the Banded Mongoose Pedigree**

**Supporting Information S1.3: Testing for signs of bias in MasterBayes paternity assignment**

**Supporting information S1.1. Microsatellite information and tests for Hardy-Weinburg equilibrium and linkage disequilibrium**

**Table S1.1.1** Details of the 43 microsatellites used in this study and (where standard multiplexes were used) the multiplex number. NAs given for loci where there was no standard multiplex and the locus was amplified either on its own or in a combination of different multiplexes.

| **Locus** | **Species Isolated From** | **Reference** | **Genbank Accession Number** | **Primer Sequence** | **Multiplex Number** |
| --- | --- | --- | --- | --- | --- |
| Mon9 | Banded mongoose (*Mungos mungo*) | N/A | KP895833 | F: TGAGCTGCCCATCATTATTGT  R: CAGGAGCTGCCTACAGACAC | 3 |
| Mon16 | Banded mongoose (*Mungos mungo*) | N/A | KP895834 | F: CCTTGGAGCAGTGAGTCCTT  R: GCTGGAATTGAGTGACAGAGC | 1 |
| Mon17 | Banded mongoose (*Mungos mungo*) | N/A | KP895835 | F: GTACAATGAAATAACATCACGG  R: CAATTTGTTCCCACTTTCAG | 1 |
| Mon19 | Banded mongoose (*Mungos mungo*) | N/A | KP895836 | F: GGGTGTCCCAGTCAGTCAGT  R: GCTCTATGTTGGCAGTGTGG | 2 |
| Mon25 | Banded mongoose (*Mungos mungo*) | N/A | KP895837 | F: ACCGCTGAAGAAATCTAGGG  R: TCGGGTGTCTGTTCAAATCTT | 1 |
| Mon29 | Banded mongoose (*Mungos mungo*) | N/A | KP895838 | F: TTGATTTTTGCTTTTGTTGA  R: TGTTTGCACTAAAAACCTCA | 3 |
| Mon31 | Banded mongoose (*Mungos mungo*) | N/A | KP895839 | F: GAGAAAAAGCACACAAATGGAGT  R: ATCTGTCTCTCTCTGTCTCTCTCTT | 3 |
| Mon32 | Banded mongoose (*Mungos mungo*) | N/A | KP895840 | F: AGGAAGTGAAGTGAGTTGTCCA  R: TCGCAATGCTTTGACAATAAG | 2 |
| Mon35 | Banded mongoose (*Mungos mungo*) | N/A | KP895841 | F: AAGGATATGACAGGCAGACC  R: CCTTCAGGGAGACATACTTCC | 3 |
| Mon36 | Banded mongoose (*Mungos mungo*) | N/A | KP895842 | F: TGATAATGGATGTCAGGCAAA  R: TGATGAAAAGCCCAAAGAGG | 3 |
| Mon38 | Banded mongoose (*Mungos mungo*) | N/A | KP895843 | F: TGAAGGCTTTGGGAGTGAAA  R: CCCATATGCTCACCCAAAAA | 2 |
| Mon41 | Banded mongoose (*Mungos mungo*) | N/A | KP895844 | F: CCCGGTTACAGACCAGTTTA  R: GGAGGAAGCAGTCTGATTTT | 1 |
| Mon42 | Banded mongoose (*Mungos mungo*) | N/A | KP895845 | F: GAAAAGGAAGAGGAGGGATA  R: ATCCTAATCATCCATACTAAAGTC | 3 |
| Mon49 | Banded mongoose (*Mungos mungo*) | N/A | KP895846 | F: ACAATGTGGTGATTTGATATGC  R: GTACATTTTGGGTGTTCTCAC | 3 |
| Mon65 | Banded mongoose (*Mungos mungo*) | N/A | KP895847 | F: TCAGAGTTTTGCTCGGAGAAG  R: TTAACTTTGATGCCCCTCCA | 2 |
| Mon66 | Banded mongoose (*Mungos mungo*) | N/A | KP895848 | F: CTCAGTCACATGGCCTTCAC  R: TGGTCTATACAGTGGGACACAGA | 2 |
| Mon67 | Banded mongoose (*Mungos mungo*) | N/A | KP895849 | F: CAGCCTGGGCTACAACTGAT  R: CTCAGAGCCTGCTGCTGTAA | 2 |
| Mon68 | Banded mongoose (*Mungos mungo*) | N/A | KP895850 | F: TGATCACAACTGAGCCAATG  R: GGATGGTATCAAGGCAAGGA | 2 |
| Mon69 | Banded mongoose (*Mungos mungo*) | N/A | KP895851 | F: GCAGTAGGTGTAAGGTGGGTCA  R: AAATTCTGCCAAGTAACATGAAAA | 1 |
| Mon70 | Banded mongoose (*Mungos mungo*) | N/A | KP895852 | F: ATGCCCTCAAAGCCTACTCA  R: GCTGAGTTATGGAAACAACCCTA | 2 |
| Ss11-12 | Meerkat (*Suricata suricatta)* | Griffin et al. 2001 | AF271118 | F: CTCATTTTCAGGAAATTTTCATCC  R: CCTAGCTTTATTTTTCTCTGTGGC | 4 |
| Ss7-1 | Meerkat (*Suricata suricatta)* | Griffin et al. 2001 | AF271115 | F: ATCCCTTAATGCATAGGCACAC  R: CCTGCTAGTCTTCTCCGTGC | N/A |
| Ss10-4 | Meerkat (*Suricata suricatta)* | Griffin et al. 2001 | AF271117 | F: CATTGGGTGCACACTGTCTC  R: CTCCAGTTCTTTTCCCTGGAG | 6 |
| Ss13-8 | Meerkat (*Suricata suricatta)* | Griffin et al. 2001 | AF271120 | F: AACAGAAGTGCCTGAATGTGC  R: TTTCCTCCACAATGAGTAAGACA | 7 |
| Mm5-1 | Banded mongoose (*Mungos mungo*) | Waldick et al. 2003 | AY142703 | F: GTTGGGCTTTGCACTG  R: GAAGAATGGACCCCTA | 4 |
| Mm10-7 | Banded mongoose (*Mungos mungo*) | Waldick et al. 2003 | AY142693 | F: CTATGAATGAAGGGGAGCAG  R: AGACAGGCTGGGTCAAAGTGA | 5 |
| TGN | Banded mongoose (*Mungos mungo*) | Waldick et al. 2003 | AY142696 | F: CTTCTCGTGTGCCAAGTCCT  R: CTGCCAGATGGGGTGACAAC | 7 |
| A248 | Banded mongoose (*Mungos mungo*) | Waldick et al. 2003 | AY155580 | F: CTACAAGATGTTTGATTATATTG  R: CAGAAGGTGTATTAATTAGCTG | 4 |
| M53 | Banded mongoose (*Mungos mungo*) | Waldick et al. 2003 | AY142700 | F: GAACACCTTTCATCACTACT  R: GCCACTATTCCAAGTCAG | 6 |
| A226 | Banded mongoose (*Mungos mungo*) | Waldick et al. 2003 | AY142694 | F: GGAGGCAGGAAATGAGATG  R: GGGTGAGGTGGCACTCTTG | 6 |
| AHT130 | Domestic dog (*Canis lupus familiaris*) | Griffin et al. 2001 | NA | F: CCTCTCCTGGTAAGTGCTGC  R: TGGAACACTGGTCCCCAG | 4 |
| Hj35 | small Asian mongoose (*Herpestes javanicus*) | Thulin et al. 2002 | AY090498 | F: AAGCCTCTATTGAGCTCTGCACT  R: TCCATATCTTCGCCAACACATT | 7 |
| Ag6 | Antarctic fur seal (*Arctocephalus gazella*) | Hoffman et al. 2008 | EU045417 | F: CCTGAGGCTCCTTCTTTCCT  R: CCAGGACCAGTGGGAAGTTA | 5 |
| Ag8 | Antarctic fur seal (*Arctocephalus gazella*) | Hoffman et al. 2008 | EU045419 | F: GGTGTGGCCTAAGAAATCCA  R: ACTGAGCTTCGGTGGAAGAC | N/A |
| Agt25 (FS15) | Antarctic fur seal (*Arctocephalus gazella*) | Hoffman & Nichols 2011 | JF746980 | F: ACTGCAGCCCTCACAACTTT  R: CAAATGCACTTTTCCCCAGT | 7 |
| Agt42  (FS41) | Antarctic fur seal (*Arctocephalus gazella*) | Hoffman & Nichols 2011 | JF746985 | F: ATCCCGGTCTTAAACCTTGC  R: TGTCATAGGTGAGGGCATGA | N/A |
| Agt44 (FS44) | Antarctic fur seal (*Arctocephalus gazella*) | Hoffman & Nichols 2011 | JF746986 | F: GAAAAAGCCACAAACCACAAA  R: GGAACTCTCCCTTTCCCTACC | 6 |
| Agt46 (FS46) | Antarctic fur seal (*Arctocephalus gazella*) | Hoffman & Nichols 2011 | ERP000497 | F: TCAAGAATGGGAAGGTGACA  R: AAACACAAACCCCCACACAT | N/A |
| Agt48 (FS48) | Antarctic fur seal (*Arctocephalus gazella*) | Hoffman & Nichols 2011 | JF746989 | F: TGCAAAACATTGTCCTCCAT  R: TGAAGTGAGCGGCTGATATTT | N/A |
| Agt50  (FS50) | Antarctic fur seal (*Arctocephalus gazella*) | Hoffman & Nichols 2011 | JF746991 | F: CAGGCTACAGCAGCTTAGGG  R: TTCGAAGAGGCTCTGGTCAT | 4 |
| Hic1-95 | Egyptian mongoose (*Herpestes ichneumon)* | Rodrigues et al. 2009 | FJ357430 | F: CGGGTAAATGCATATAGC  R: GCGTTCCTTTTACAGCAT | N/A |
| Hic2-52 | Egyptian mongoose (*Herpestes ichneumon)* | Rodrigues et al. 2009 | FJ357432 | F: AAACCTGCTTGGGATTCG  R: ACTGTCTCGGTGTTAGTC | 5 |
| Hic4-30 | Egyptian mongoose (*Herpestes ichneumon)* | Rodrigues et al. 2009 | FJ357438 | F: GAAATTGAGGCTTCCATG  R: AGAGTGTGGAAACAGGAC | 5 |

**S1.1.2 PCR conditions per 12µl reaction**

| **Volume** | **Component** |
| --- | --- |
| 6µl | 2 x Qiagen reaction mix |
| 1.2µl | 10 x primer mix |
| 1.8µl | RNAse free water |
| 3µl | Template DNA |

**S1.1.3 PCR Cycle**

| **Step** | **Time** | **Temperature (˚C)** | **Cycles** |
| --- | --- | --- | --- |
| Activation | 15 min | 95 | 1 |
| Denaturation | 30 s | 94 | 35 |
| Annealing | 90 s | 57 |  |
| Extension | 60 s | 72 |  |
| Final Extension | 30 min | 60 | 1 |

**S1.1.4** Allele information and probabilities of deviation from Hardy-Weinburg equilibrium calculated using Genepop 4.3 (Raymond & Rousset, 1995; Rousset 2008). The number of alleles (k), the number of individuals genotyped at that loci (N), goodness of fit to Hardy-Weinburg equilibrium (*P*(HW)), and the frequency of null alleles (F(Null); according to Weir & Cockerham, 1984 (W&C) and Robertson and Hill, 1984 (R&H)) from genotype data of 1787 banded mongooses; proportion of Hardy-Weinburg Exact tests (Prop HW) carried out on 300 randomised subpopulations containing 21 individuals from different social groups (i.e. non-relatives) where locus did not fit Hardy-Weinburg equilibrium under P < 0.05, and controlling for false detection rates following Benjamini and Hochberg (1995) (FDR).

| **Locus** | **k** | **N** | ***P*(HW)** | **F(Null)** | | **Prop HW** | |
| --- | --- | --- | --- | --- | --- | --- | --- |
|  |  |  |  | **W&C** | **R&H** | **P < 0.05** | **FDR** |
| Mon16 | 6 | 1777 | 0.00 | 0.05 | 0.04 | 0.07 | 0.02 |
| Mon17 | 4 | 1779 | 0.05 | 0.01 | 0.02 | 0.07 | 0.02 |
| Mon25 | 8 | 1779 | 0.00 | 0.07 | 0.03 | 0.12 | 0.06 |
| Mon41 | 3 | 1776 | 0.16 | -0.01 | -0.01 | 0.02 | 0.01 |
| Mon69 | 9 | 1774 | 0.00 | 0.03 | 0.05 | 0.06 | 0.03 |
| Mon19 | 6 | 1778 | 0.00 | 0.01 | 0.01 | 0.09 | 0.05 |
| Mon32 | 3 | 1778 | 0.67 | 0.02 | 0.01 | 0.02 | 0.01 |
| Mon38 | 5 | 1772 | 0.00 | -0.05 | 0.00 | 0.06 | 0.03 |
| Mon65 | 2 | 1779 | 0.96 | 0.00 | 0.00 | 0.02 | 0.01 |
| Mon66 | 2 | 1781 | 0.00 | 0.11 | 0.11 | 0.07 | 0.06 |
| Mon67 | 4 | 1780 | 0.35 | -0.03 | -0.01 | 0.01 | 0.01 |
| Mon68 | 4 | 1771 | 0.00 | 0.08 | 0.04 | 0.11 | 0.07 |
| Mon70 | 5 | 1774 | 0.00 | 0.05 | 0.04 | 0.06 | 0.04 |
| Mon29 | 2 | 1760 | 0.00 | 0.10 | 0.10 | 0.04 | 0.03 |
| Mon31 | 5 | 1779 | 0.00 | -0.04 | 0.00 | 0.04 | 0.02 |
| Mon35 | 4 | 1774 | 0.01 | 0.00 | 0.04 | 0.04 | 0.01 |
| Mon36 | 4 | 1777 | 0.00 | 0.03 | 0.03 | 0.07 | 0.03 |
| Mon42 | 5 | 1777 | 0.00 | 0.04 | 0.03 | 0.07 | 0.03 |
| Mon49 | 4 | 1780 | 0.01 | 0.02 | 0.02 | 0.07 | 0.03 |
| Mon9 | 7 | 1766 | 0.00 | 0.07 | 0.03 | 0.14 | 0.08 |
| A226 | 3 | 1746 | 0.03 | 0.06 | 0.03 | 0.02 | 0.00 |
| A248 | 4 | 1706 | 0.07 | 0.05 | 0.02 | 0.02 | 0.01 |
| Ag6 | 5 | 1588 | 0.00 | 0.05 | 0.07 | 0.05 | 0.03 |
| Hj35 | 10 | 1533 | 0.00 | 0.07 | 0.04 | 0.07 | 0.04 |
| M53 | 4 | 1727 | 0.01 | 0.01 | 0.06 | 0.02 | 0.01 |
| Mm10-7 | 3 | 1703 | 0.12 | 0.04 | 0.03 | 0.06 | 0.03 |
| Mm5-1 | 3 | 1725 | 0.10 | 0.03 | 0.02 | 0.03 | 0.01 |
| ss10-4 | 5 | 1706 | 0.00 | 0.10 | 0.15 | 0.12 | 0.06 |
| ss13-8 | 7 | 1732 | 0.00 | 0.01 | 0.02 | 0.08 | 0.02 |
| TGN | 6 | 1725 | 0.00 | 0.05 | 0.04 | 0.07 | 0.02 |
| FS1 | 2 | 1681 | 0.00 | -0.08 | -0.08 | 0.05 | 0.01 |
| fs4 | 4 | 1664 | 0.00 | -0.01 | 0.05 | 0.01 | 0.00 |
| fs4 | 2 | 1337 | 0.00 | -0.13 | -0.13 | 0.01 | 0.00 |
| fs4 | 3 | 978 | 0.00 | 0.09 | 0.24 | 0.01 | 0.00 |
| fs5 | 3 | 1553 | 0.77 | 0.02 | 0.02 | 0.04 | 0.01 |
| hic2.5 | 8 | 1675 | 0.04 | 0.03 | 0.01 | 0.08 | 0.04 |
| hic4.3 | 8 | 1502 | 0.00 | 0.11 | 0.10 | 0.11 | 0.07 |
| Ss11-12 | 9 | 1724 | 0.00 | 0.11 | 0.04 | 0.07 | 0.05 |
| AHT130 | 3 | 1602 | 0.49 | -0.03 | -0.02 | 0.07 | 0.07 |
| Ag8 | 3 | 1356 | 0.16 | 0.04 | 0.04 | 0.06 | 0.05 |
| Ss7-1 | 5 | 1279 | 0.00 | 0.09 | 0.07 | 0.06 | 0.05 |
| fs4 | 7 | 421 | 0.00 | 0.17 | 0.17 | 0.06 | 0.04 |
| hic1.9 | 5 | 587 | 0.00 | -0.01 | 0.02 | 0.02 | 0.01 |

**S1.1.5** Summary of probabilities of linkage disequilibrium calculated using Genepop 4.3 (Raymond & Rousset, 1995; Rousset 2008) on 300 randomised subpopulations containing 21 individuals from different social groups (i.e. non-relatives): the proportion of tests significant at the P < 0.05 level; the proportion of tests significant after controlling for false detection rates (FDR) within each test following Benjamini and Hochberg (1995). There were 43 loci giving a total 903 tests for linkage disequilibrium in each randomised subpopulation. No pairs of loci were consistently significant across randomisations.

| **Proportion of 300 test results significant and suggestive of linkage disequilibrium** | **P < 0.05** | **FDR**  **(*P* = 0.00 – 0.049)** |
| --- | --- | --- |
| < 5% | 628 | 870 |
| ≥ 5%, < 10% | 245 | 30 |
| ≥ 10%, < 20% | 27 | 1 |
| ≥ 20%, < 30% | 1 | 1 |
| ≥ 30%, < 40% | 0 | 1 |
| ≥ 40%, < 50% | 1 | 0 |
| >50 % | 0 | 0 |

**Supporting Information S1.2: Construction of the Banded Mongoose Pedigree**

A pedigree for the Banded Mongoose Research Project (BMRP) study population was recovered by combining genotypic data and phenotypic information in two different programs (MasterBayes and Colony2). Genotypic data was available at (up to) 43 microsatellite loci for 1786 individuals from a total of 2878 individuals observed in the study population. The inferred 9-generation deep pedigree includes 1491 maternities and 1426 paternities with known identity and at high confidences. Additional to this, where Colony2 inferred sibships at high confidence within a subset of founders and immigrants a further 71 dummy maternities and 78 dummy paternities were assigned. These additional dummy parentages provided information about the relatedness between founders and immigrants within the population, adding depth to the pedigree and enhancing estimates of relatedness and inbreeding coefficients.

Genotypic data

Preparation of genotype database

In total, 1822 tissue samples were genotyped using a 43-loci multiplex kit (blood and tissue samples collected between 1996 and 2014). This dataset was put through a quality check to remove erroneous samples highlighted through (1) mismatching duplicate samples, (2) identified genotype matches (i.e. where an individual was sampled twice under different identities) and (3) genotype sample identities not matching individual identities in life history database. Summaries of edits to genotype data are given below. Following these edits genotypic data was available for a total of 1787 individuals.

Genotyping error rates

Per locus genotyping error rates were manually calculated from the proportion of mismatching genotypes for any samples that were genotyped two or more times following Hoffman & Amos (2005). Error rates were calculated separately with errors attributable to (i) allelic dropout (class I error) and (ii) stochastic typing errors (class II error). Note that where error rates were calculated as zero they were input as 0.005 in both MasterBayes and Colony2 analyses because it is unlikely that genotyping at any loci can be completely free from error.

Table S1.2.1 Estimated genotyping error rates

| **locus** | **total alleles re-typed** | **total error rate (per allele)** | **allelic dropout rate** | **other error rate** |
| --- | --- | --- | --- | --- |
| Mon16 | 128 | 0.0000 | 0 | 0 |
| Mon17 | 128 | 0.0000 | 0 | 0 |
| Mon19 | 128 | 0.0000 | 0 | 0 |
| Mon25 | 128 | 0.0000 | 0 | 0 |
| Mon29 | 128 | 0.0000 | 0 | 0 |
| Mon31 | 128 | 0.0000 | 0 | 0 |
| Mon32 | 128 | 0.0000 | 0 | 0 |
| Mon35 | 128 | 0.0000 | 0 | 0 |
| Mon36 | 128 | 0.0000 | 0 | 0 |
| Mon38 | 128 | 0.0000 | 0 | 0 |
| Mon41 | 128 | 0.0000 | 0 | 0 |
| Mon42 | 128 | 0.0000 | 0 | 0 |
| Mon49 | 128 | 0.0000 | 0 | 0 |
| Mon65 | 128 | 0.0000 | 0 | 0 |
| Mon66 | 128 | 0.0000 | 0 | 0 |
| Mon67 | 128 | 0.0000 | 0 | 0 |
| Mon68 | 128 | 0.0000 | 0 | 0 |
| Mon69 | 128 | 0.0078 | 0.0078125 | 0 |
| mon70 | 128 | 0.0000 | 0 | 0 |
| Mon9 | 128 | 0.0000 | 0 | 0 |
| FS15 | 1720 | 0.0209 | 0.019186047 | 0.001744186 |
| FS44 | 1684 | 0.0018 | 0 | 0.001781473 |
| FS46 | 1570 | 0.0274 | 0.02611465 | 0.001273885 |
| FS50 | 1304 | 0.0199 | 0 | 0 |
| FS41 | 54 | 0.0556 | 0.055555556 | 0 |
| FS48 | 16 | 0.1250 | 0.125 | 0 |
| Hic2.52 | 1836 | 0.0142 | 0.010348584 | 0.003812636 |
| Hic4.30 | 1248 | 0.0160 | 0.014423077 | 0.001602564 |
| Hic1.95 | 26 | 0.0000 | 0 | 0 |
| ss11-12 | 188 | 0.0106 | 0.010638298 | 0 |
| ss7-1 | 184 | 0.0000 | 0 | 0 |
| ss10-4 | 168 | 0.0060 | 0.005952381 | 0 |
| ss13-8 | 180 | 0.0000 | 0 | 0 |
| mm5-1 | 184 | 0.0000 | 0 | 0 |
| mm10-7 | 160 | 0.0000 | 0 | 0 |
| TGN | 164 | 0.0244 | 0.018292683 | 0.006097561 |
| a248 | 176 | 0.0057 | 0.005681818 | 0 |
| m53 | 180 | 0.0111 | 0.011111111 | 0 |
| a226 | 180 | 0.0056 | 0.005555556 | 0 |
| aht130 | 160 | 0.0000 | 0 | 0 |
| hj35 | 168 | 0.0119 | 0.005952381 | 0.005952381 |
| ag6 | 172 | 0.0058 | 0.005813953 | 0 |
| ag8 | 160 | 0.0063 | 0.00625 | 0 |

Parentage Inference

Familial relationships within the BMRP study population were inferred using field observations, individual genotypes, and two freely available programs; MasterBayes v2.51 (http://cran.r-project.org/), which was implemented in R v3.1.1, and Colony 2.0.5.7 (<http://www.zsl.org/science/software/colony>).

BMRP Dataset

The BMRP dataset included 2878 individuals observed on the Mweya Peninsular between 1996 and 2014. Of these 2878 individuals (1787 genotyped individuals), the birth date and birth pack was known for 2633 individuals (1593 genotyped individuals); these individuals were classified as ‘offspring’ within parentage assignment programs with candidate parents inferred using field observations (see below).

MasterBayes

MasterBayes genotypic data

A text file of the edited genetic dataset was provided to MasterBayes for inclusion in the analysis. Estimated genotyping error rates were used to set per locus error rates.

MasterBayes phenotypic data

All individuals in the BMRP were included in the MasterBayes model with each individual attributed an identifier as to whether or not it was an offspring (1) and thus required parentage assignment, or a potential parent (0) in any given month during the duration of the project. Any individual born into the study population was included in the dataset as an offspring (1) in the month that it was born and then as a (0) in every month until it died or left the study population. Individuals that were not born into the study population (i.e. they were either immigrants or founders) were included with a (0) in every month that they were seen within the study population.

The following variables were also included as phenotypic information for each month that individuals were known to be alive within the study population: sex (male/female/unknown), age (in months), social group (46 different social groups), whether or not a female was observed giving birth (0/1).

MasterBayes program parameters and settings

The following restrictions were applied in the MasterBayes analysis to limit candidate mothers and fathers:

- Female in offspring pack in birth month
- Juveniles (individuals < 6 months old) can’t be parents
- Offspring can’t be their own parents
- Mother have to be alive in birth month
- Father have to be alive in month 2 months prior to birth (conception month)

The following phenotypic variables were also included in the MasterBayes analysis to assist with parentage assignment:

- Female recorded as given birth in month of, or either side of birth month
- Male recorded in same pack as offspring in 3 months prior to birth (note: this time frame differs to the restriction of males being alive to allow for occasions where group formation or split meant that pack membership was temporarily dynamic and could be inaccurate)
- Female quadratic age (months)
- Male quadratic age (months)

The Markov chain Monte Carlo (MCMC) estimation chain was run for 1,500,000 iterations with a thinning interval of 500, and a burn-in of 500,000. No further prior distributions were specified and default improper priors were used. Metropolis–Hastings acceptance rates were checked to be within the correct rage (0.2 and 0.5) and hence no tuning parameter was used.

Samples of the posterior probability density distributions for parentage assignments and phenotypic predictors or parentage were returned. Confidence in parentage assignment was calculated as the proportion of iterations for which an individual was assigned parentage to a particular offspring. Individuals were considered as parents for downstream pedigree analyses if they were assigned with at least 80% confidence (marginal probability).

Model code implemented in R:

##restrictions

res1<-expression(varPed(x="packs",gender="Female",relational="OFFSPRING", restrict="==")) #females within same pack in same month

res2<-expression(varPed(x="juv", restrict=FALSE)) #juveniles can't be

parents

res3<-expression(varPed(x="id", relational="OFFSPRING",restrict="!="))

#offspring can't be parents

res4<-expression(varPed(x="datesalive",gender="Female",

relational="OFFSPRING", restrict="=="))

res5<-expression(varPed(x="datesalive",gender="Male",

relational="OFFSPRING", restrict=(-2)))

##variables

var1<-expression(varPed(x="givenbirth",gender="Female",lag=c(-1,1)))

var2<-expression(varPed(x="packs", gender="Male", relational="OFFSPRING",

lag=c(-3,0))) # paternal variable: group membership of candidate males in the 3 months prior to offspring birth

var3<-expression(varPed(x="agemM",gender="Female"))

var4<-expression(varPed(x="agemM",gender="Male"))

var5<-expression(varPed(x="agemsq",gender="Female"))

var6<-expression(varPed(x="agemsq",gender="Male"))

PdP<-PdataPed(list(res1, res2, res3,res4,res5, var1, var2,var3,var4,var5,var6),data=P,USsire=TRUE,USdam=TRUE)

##genotype data

GdP<-GdataPed(G, perlocus=TRUE) ##nb has per-locus error rates

sP<-startPed(estG=FALSE, E1=error$E1, E2=error$E2)

# use the specified per locus error rates

pP<-priorPed(beta=list(mu=c(0,0,0,0,0,0), sigma=diag(c(rep(1+pi^2/3,6))))) ##uninformative prior for 6 variable model

model<-MCMCped(PdP,GdP, sP=sP, pP=pP, write_postP="JOINT", DSapprox=TRUE,

jointP=FALSE,

nitt=1500000, thin=500, burnin=500000,

verbose=T)

Colony2

Colony2 can be used to infer sibship groups within candidate offspring in the absence of any candidate parents. In the BMRP dataset this could be used to estimate sibships within groups of founder and/or immigrant individuals which can add depth to the pedigree and enhance estimates of relatedness and inbreeding coefficients. Therefore all individuals with genotypic data were included as candidate offspring in the Colony2 model to allow for the estimation of sibship groups.

Colony2 dataset

Candidate parent lists were generated with the same criteria as in the MasterBayes analyses. Specifically, candidate maternal and paternal identities were extracted from the MasterBayes model output using ‘X.list$X[[i]]$restdam.id’ and ‘X.list$X[[i]]$restsire.id’, respectively, and then used to generate exclusion lists for input into Colony2. By definition, founder and immigrant individuals had no candidate parents and thus all maternal and paternal identities were included in exclusion lists.

Colony2 parameters and settings

The probability that the true mother and father were in the candidate lists were both set as 0.8. No Maternal or paternal sibships were excluded. Both male and female mating systems were set as ‘polygamous’. A weak sibship prior was included as 1.5 for both maternal and paternal sibships to limit false-positive sibship assignments. The model was run with ‘no scaling’ as a preliminary run with scaling recovered spurious sibship groups containing hundreds of offspring. Allele frequencies and per locus error rates were set as those estimated from Cervus and those calculated from repeated genotyping, respectively. Parentage assignments with at least 80% individual-level confidence were considered in the final combination of parentage results.

Program Results

MasterBayes

MasterBayes assigned confident maternity and paternity to 1490 and 1397 offspring, respectively (assignment rates of 94% and 88%). Overall, paternity assignment probability was high with 88% of genotyped offspring being assigned a father with a confidence of >= 0.95.

Assignment rates were higher post- year 2000, likely because of both an increased genotypic sampling effort and more accurate phenotypic data (see below; figure 1). All autocorrelations were checked and found to be < 0.01.

All phenotypic predictors were found to be predictors of parentage. The following coefficients were extracted for each variable as the posterior mode and highest posterior density (HPD) intervals from posterior densities:

| Table S1.2.2 Posterior modes and highest posterior density distrobutions for phenotypic predictors of parentage in MasterBayes | | | |
| --- | --- | --- | --- |
|  | Posterior Mode | **HPD interval** | |
| ♀ Given Birth | 6.18 | 5.61 | 6.83 |
| ♀ Age (months) | 0.42 | 0.35 | 0.50 |
| ♀ Age^2 (months) | -0.017 | -0.020 | -0.014 |
| ♂ Same Pack | 3.85 | 3.72 | 4.03 |
| ♂ Age (months) | 0.89 | 0.82 | 0.94 |
| ♂ Age^2 (months) | -0.033 | -0.036 | -0.031 |


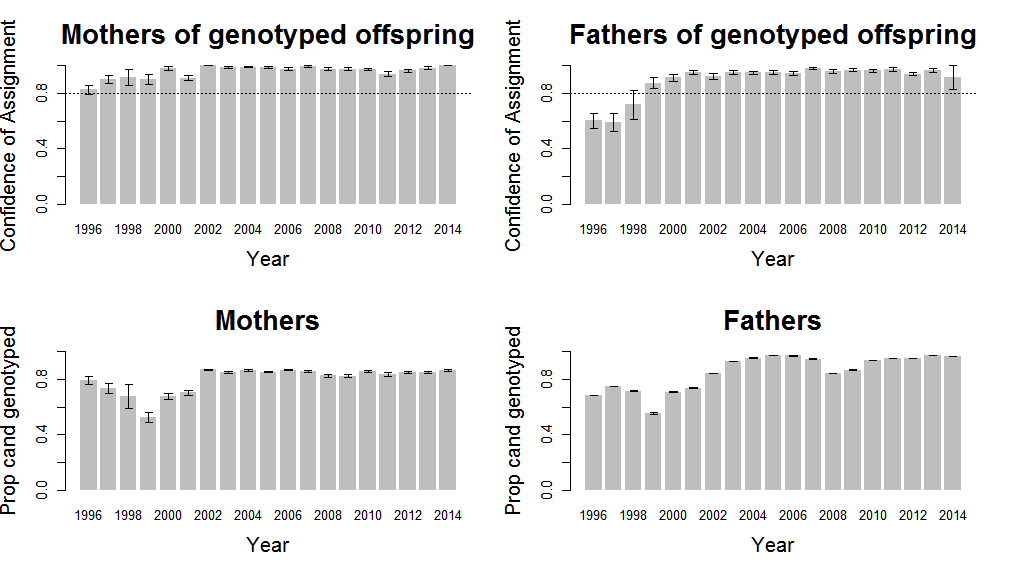


Figure S1.2.1. Confidence of MasterBayes assignment and the proportion of candidate parents genotyped per offspring by year from 1996 to 2014. Bars and error bars give mean values and standard errors, respectively

MasterBayes estimates of the number of unsampled dams and sires (note that here ‘unsampled’ refers to individuals that did not appear in the phenotypic dataset rather than ungenotyped individuals):

| Table S1.2.3 Posterior modes and highest posterior density distributions of the number of unsampled sires and dams estimated by MasterBayes | | | |
| --- | --- | --- | --- |
|  | Posterior Mode | HPD interval | |
| Sires | 2.39 | 1.46 | 3.46 |
| Dams | 0.17 | 0.12 | 0.27 |

The number of mismatched alleles between offspring and assigned parents was not limited in MasterBayes assignment. The number of mismatching alleles between offspring and parents assigned with >0.8 confidence was:

Table S1.2.4 Number of confidently assigned parents with

mismatching alleles

| Number of mismatching alleles | 0 | 1 | 2 | 3 | 4 |
| --- | --- | --- | --- | --- | --- |
| Dam | 1320 | 102 | 21 | 4 | 3 |
| Sire | 1262 | 112 | 15 | 4 | 1 |

Colony2

Colony2 returns a list of the most likely Maternity and Paternity identities (with associated individual-level likelihoods) as well as full- and half-sib dyads (again with associated individual-level likelihoods) and a Best Maximum Liklihood Configuration (Best(ML) Config) for each offspring. This Best (ML) Config summarizes ‘dummy’ parents assigned to offspring that are in estimated sibship groups.

Colony assigned 1200 maternities and 1029 paternities of known identity with a likelihood of at least 0.8. These assignments were used to assign paternity and maternity to 29 and 45 offspring where MasterBayes failed to confidently assign parentage, respectively.

Colony also assigned 5659 full-sib dyads and 32484 half-sib dyads with a likelihood of at least 0.8. Where Colony had confidently assigned full -sibships the ‘dummy’ parents from the (Best(ML) Config) were used to assign parentage under certain criteria (see below).

Comparison of MasterBayes and Colony2 parentage assignments

| Table S1.2.5 Number of offspring assigned parentage above and below a confidence threshold of 0.8 in MasterBayes and Colony | | | |
| --- | --- | --- | --- |
|  |  | Maternity | Paternity |
| MasterBayes assigned  ≥ 0.8 | Total | 1474 | 1397 |
|  | Colony assigned ≥ 0.8; matching | 1116 | 915 |
|  | Colony assigned ≥ 0.8; mismatched | 55 | 69 |
|  | Colony assigned < 0.8 | 303 | 413 |
| Colony assigned  ≥ 0.8 | Total | 1200 | 1029 |
|  | MasterBayes assigned ≥ 0.8; matching | 1116 | 915 |
|  | MasterBayes assigned ≥ 0.8; mismatched | 55 | 69 |
|  | MasterBayes assigned < 0.8 | 29 | 45 |
| MasterBayes & Colony both < 0.8 | | 1375 | 1436 |

Combination of program results

We used a series of 4 rules to assign parentage:

***Direct parentage assignments***

**1. MasterBayes Assignment**

MasterBayes was able to assign a lot more confident parentages than Colony2, likely because of MasterBayes was able to use phenotypic data to assign parentages more accurately. We therefore used all confident MasterBayes parentages to assign parentages including whenever MasterBayes and Colony2 confidently assigned mismatching parents.

**2. Colony Assignment**

Confident Colony2 parentage assignments were used wherever MasterBayes failed to confidently assign parentage.

***Parentage assignments inferred by Colony2 sibships***

Colony2 is able to assign sibships in the absence of any candidate parents allowing the assignment of ‘dummy’ parents to individual in sibship groups. Limiting this ‘dummy’ parent dataset to ‘dummy’ parents inferred from confident full-sibships we assigned paternity/maternity under the following rules (NB the same rules were followed for both maternity and paternity assignment but only maternity rules are listed here for ease of understanding):

**3. Assignment of MasterBayes maternity inferred through Colony sibships**

**(a)** Where all siblings within the sibship with maternity assigned from MasterBayes were assigned the same mother AND all siblings within the sibship that were not assigned maternity from MasterBayes were also NOT offspring (i.e. founders and/or immigrants) the MasterBayes maternal identity was assigned to all siblings. (N.B. in one case this resulting in an individual being assigned as its own father – that individual [W3] was assigned no paternal identity)

**(b)** Where all siblings within the sibship assigned maternity from MasterBayes were assigned the same mother AND one or more siblings within the sibship that were not assigned maternity from MasterBayes were offspring (i.e. individuals with candidate parent lists) the sibship was considered to be in disagreement with MasterBayes assignments and so unassigned siblings within the sibship were left unassigned. (N.B. if they were true full-sibships we would expect MasterBayes to assign the same parent to all siblings.)

**(c)** Where siblings within the sibship with maternity assigned from MasterBayes were assigned to more than one mother the sibship was considered to be in disagreement with MasterBayes and so unassigned siblings within the sibship were left unassigned.

**4. Assignment of dummy maternity inferred through Colony sibships**

Where no sibling within the sibship was assigned maternity from MasterBayes the ‘dummy’ maternity was assigned to all siblings.

| Table S1.2.6 Number of offspring assigned maternity and paternity identities following the 4 assignment rules outlined in the main text | | |
| --- | --- | --- |
| Assignment Rule | Maternity | Paternity |
| 1. Confident MasterBayes assignment | 1474 | 1397 |
| 2. Confident Colony2 assignment | 29 | 45 |
| 3. Identity inferred using Colony2 sibship and MasterBayes parentage | 34 | 19 (after 1 removed because assigned as own father) |
| 4. Dummy identity inferred using Colony2 sibship | 33 | 15 |

The BMRP Pedigree

A pedigree data frames (‘id’, ‘dam’, ‘sire’) was generated from a list of individuals with at least one assigned parent. This data frame was then edited using the ‘fixPedigree’ function in the R package pedantics (this generates a data frame where all individuals appear as offspring in lines above where they appear as parents).

Pedigree statistics

Pedigree summary statistics (including pairwise relatedness and inbreeding coefficients) were calculated for both pedigrees using the ‘pedigreeStats’ function in the R package ‘pedantics’ v1.01.

| Table S1.2.7 Summary statistics from the Banded Mongoose Research Project pedigree | |
| --- | --- |
| Statistic | Banded Mongoose Project Pedigree |
| Records | 1748 |
| Maternities | 1570 |
| Paternities | 1476 |
| Full sibs | 3595 |
| Maternal sibs | 13589 |
| Maternal half sibs | 9994 |
| Paternal sibs | 12598 |
| Paternal half sibs | 9003 |
| Maternal grandmothers | 1174 |
| Maternal grandfathers | 947 |
| Paternal grandmothers | 1078 |
| Paternal grandfathers | 895 |
| Maximum pedigree depth | 9 |
| Mean maternal sibship size | 7.1 |
| Mean paternal sibship size | 7.0 |


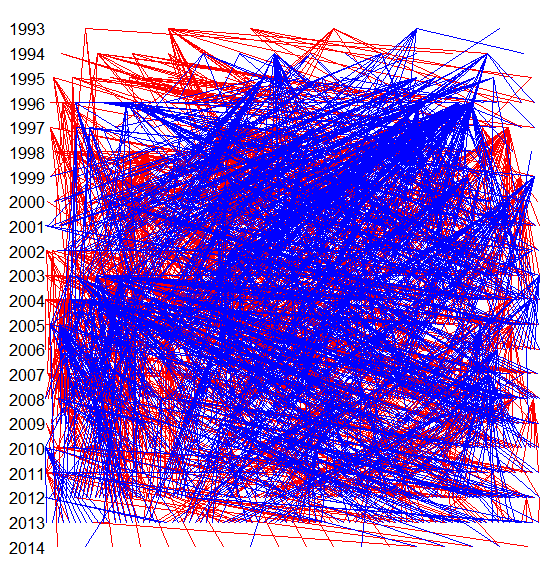


Figure S1.2.2. The Banded Mongoose Project Pedigree. Red lines show maternal links and blue lines show paternal links.

**Supporting Information S1.3: Testing for signs of bias in MasterBayes paternity assignment**

Overall, MasterBayes paternity assignment probability was high with 91% and 88% of genotyped offspring being assigned a father with a confidence of >= 0.8 and >= 0.95, respectively.


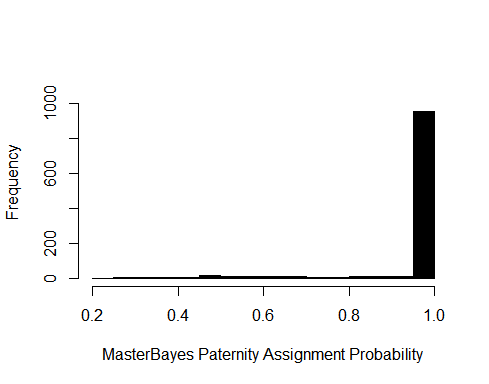


Figure S1.3.1 Histogram of confidence for paternity assignment of 1083 pups assigned using MasterBayes.

However, with 97 (from a total of 1083) pups remaining unassigned by MasterBayes (i.e. with a probability under our acceptance threshold of 0.8) it remains possible that assignment bias towards unrelated mating pairs may be affecting our results.

Paternity assignment may occur at low confidence if (i) the mating pair is highly related and/or (ii) the real father is not sampled/genotyped. Of the 97 pups with < 0.8 confidence of paternity assignment in MasterBayes, 32 were assigned to known males which were not genotyped, 26 were assigned to unsampled fathers (i.e. paternity assigned as ‘NA’ which means the father did not appear in the genotype or phenotype data; note that no pups were confidently assigned to an unsampled father) and 39 were assigned to genotyped fathers. This suggests that the biggest cause of low assignment confidence within our dataset is missed sampling of a small sample of candidate fathers (34/492 candidate fathers included in the MasterBayes analyses were not genotyped).

To test if high relatedness between mating pairs contributed to the low confidence of paternity assignment within our dataset we fitted the marginal paternity assignment probability as the response term in a GLMM with the following variables as fixed predictors:

1. Number of within-group males
2. Number of within-group males that were not genotyped
3. Pedigree-derived relatedness between assigned mother and father
4. Mean pedigree-derived relatedness of assigned father to other within-group males
5. Mean male-male pedigree-derived relatedness of all within-group males
6. Mean pedigree-derived relatedness of assigned father to within-group females
7. Mean male-female pedigree-derived relatedness for all individuals within the group

This analysis allowed us to investigate if the likelihood of confidently assigning paternity varied with group variation in relatedness structure (v, vii), individual relatedness to other members of the group (iv, vi) and/or variation in relatedness for specific mating events (iii). These analyses were limited to 906 estimates of paternity assignment confidence of a genotyped offspring assigned to a sampled father. We also fitted social group, litter, mother, and father identities as random effects to control for repeated measures (note that fitting random effects in this way also allows us to test if confidence of paternity assignment varies across groups, litters, and individuals).

We found paternities to be assigned at significantly lower confidence within mating pairs that were more related (GLMM; χ^2^_(1)_ = 14.01, p < 0.001; figure SI3.2). Confidence of paternity assignment was also decreased when there was a larger number of within-group candidate males that were not genotyped (GLMM; χ^2^_(1)_ = 7.31, p = 0.007; figure SI3.3). None of the other variables tested were found to have significant effects on confidence of paternity assignment (table SI3.1), though there was a tendency for confidence of paternity assignment to be decreased when there were more candidate males within the group (GLMM; χ^2^_(1)_ = 3.20, p = 0.073). We also found no evidence that confidence of paternity assignment varied between groups, litters, fathers or mothers (table SI3.2)

| Table S1.3.1 Factors affecting variation in confidence of paternity assignment in MasterBayes | | | | | |
| --- | --- | --- | --- | --- | --- |
| **Explanatory terms** | **Effect Size ± SE** | | | **χ^2^** | **p** |
|  |  | | |  |  |
| Number of within-group males | 0.001 | ± | 0.001 | 3.20 | 0.073 |
| **Number of within-group males that were not genotyped** | **-0.007** | **±** | **0.002** | **7.31** | **0.007** |
| **Relatedness between assigned mother and father** | **-0.08** | **±** | **0.02** | **14.01** | **< 0.001** |
| Mean relatedness of assigned father to other within-group males | 0.04 | ± | 0.04 | 1.36 | 0.24 |
| Mean male-male relatedness of all within-group males | 0.07 | ± | 0.06 | 1.54 | 0.21 |
| Mean relatedness of assigned father to within-group females | 0.02 | ± | 0.04 | 0.43 | 0.51 |
| Mean male-female relatedness for all individuals within the group | 0.001 | ± | 0.06 | < 0.001 | 0.99 |
| Constant | 0.98 | ± | 0.01 |  |  |
| Coefficient estimates and p values from a GLMM with 960 estimates of paternity assignment confidence from 191 litters, 14 social groups, 167 fathers and 167 mothers. | | | | | |

| Table S1.3.2 Variance estimates from a GLMM estimating the effects of within-group relatedness structure on the confidence of MasterBayes paternity assignment | | | |
| --- | --- | --- | --- |
| **Random effect** | **Variance Estimate ± SE** | | |
|  |  | | |
| Social group | 0.0005 | ± | 0.057 |
| Litter | 0.0001 | ± | 0.012 |
| Father identity | 0.0044 | ± | 0.067 |
| Mother identity | 0.0006 | ± | 0.024 |
| Residual | 0.0033 | ± | 0.058 |
| Variance estimates from a GLMM with 960 estimates of paternity assignment confidence from 191 litters, 14 social groups, 167 fathers and 167 mothers. | | | |


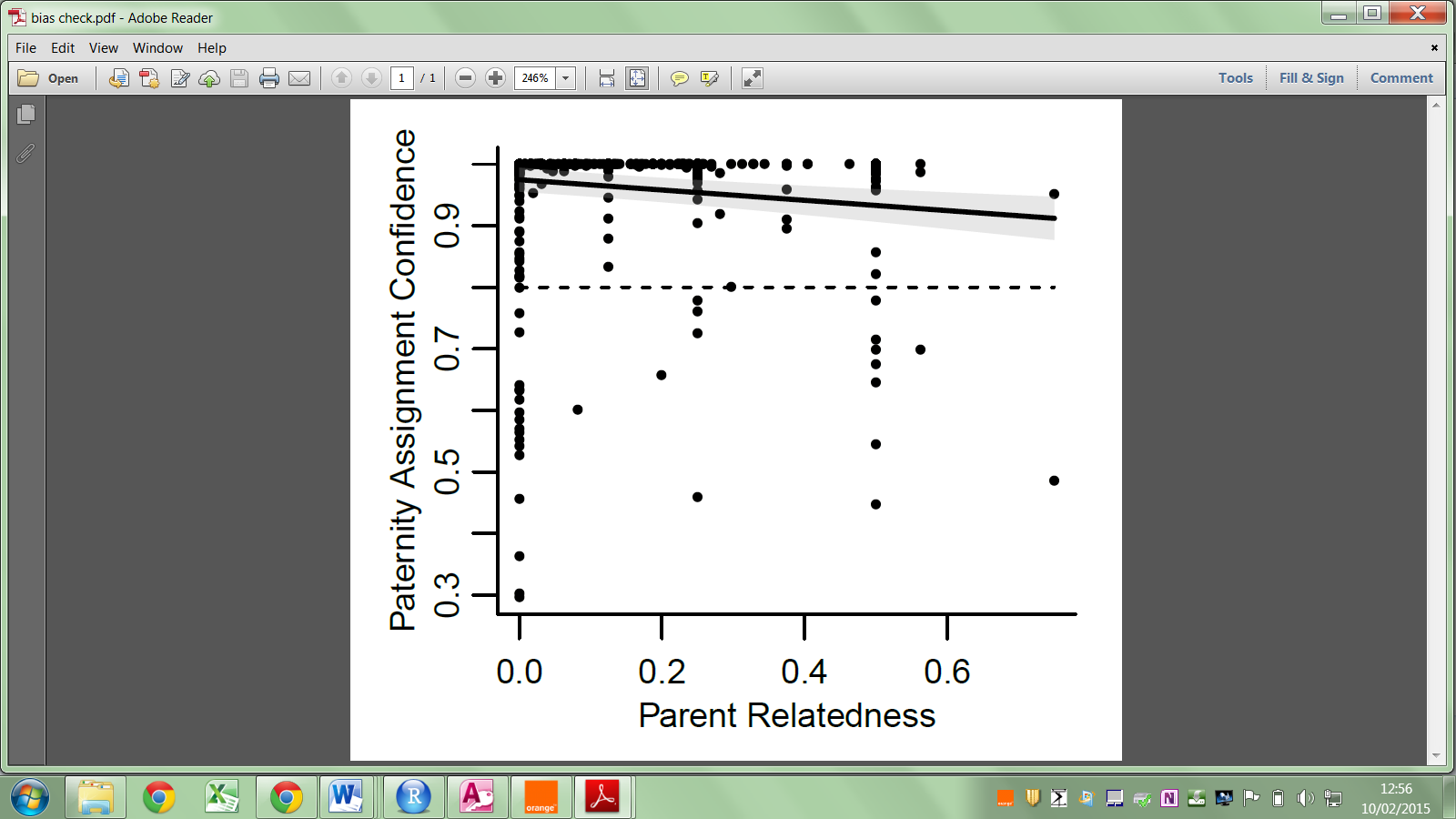


Figure S1.3.2 Effect of parent relatedness on the confidence of paternity assignment in MasterBayes. Dots show raw values, line and shaded area show predicted trend with standard error calculated from a GLMM while controlling for a significant effect of the number of within group males which were not genotyped (table S1.3.1). Dotted line shows the 0.8 threshold for acceptance of paternity assignment used in this study.


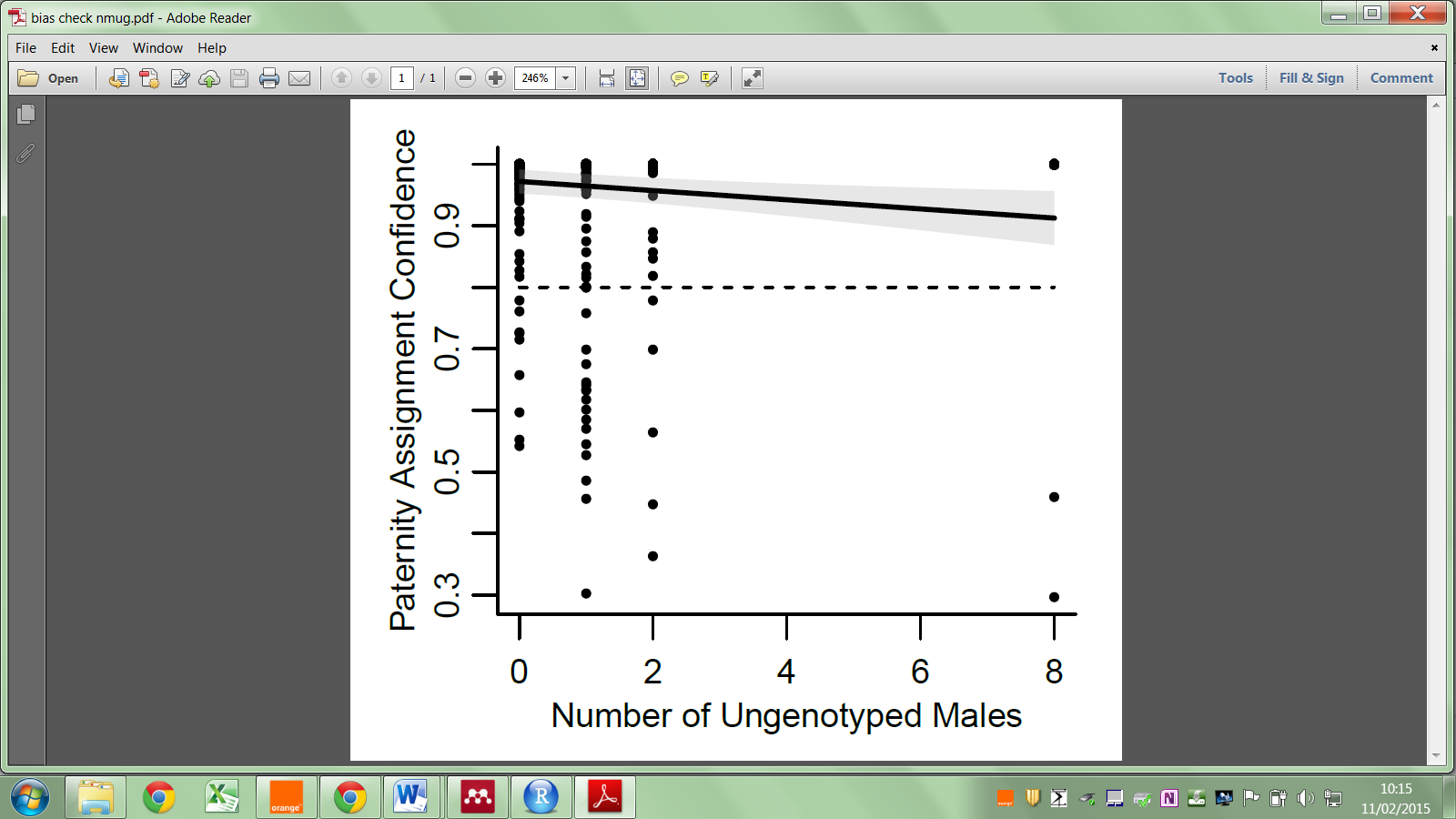


Figure S1.3.3 Effect of the number of within-group candidate fathers which were not genotyped on the confidence of paternity assignment in MasterBayes. Dots show raw values, line and shaded area show predicted trend with standard error calculated from a GLMM while controlling for a significant effect of parent relatedness (table S1.3.1).

**Conclusions**

Here, we have investigated the possibility of bias in MasterBayes paternity assignment towards unrelated mating partners which may generate a pedigree indicative of inbreeding avoidance in the absence of any real mating preferences towards less related mating partners.

MasterBayes was able to confidently assign (> 0.8) paternity to a large proportion of the genotyped offspring which were included in the assignment models. This high confidence of paternity assignment is likely to be attributable to the fact that 93% of candidate fathers included in the paternity assignment were genotyped and the large number of microsatellite markers (43; see S1.1) used to assign parentage.

We found 33% and 27% of offspring with unconfidently assigned paternity to be assigned to males that were not genotyped and unsampled males (i.e. males that were not recorded within the study population), respectively. This suggests that the largest contributor to assignment of paternity with low confidence was missed sampling of a small proportion of candidate males.

An analysis into the factors driving low confidence of assignment in offspring assigned to genotyped fathers found that confidence of paternity assignment was significantly lower when there were more candidate males within the group that were not genotyped. This result suggests that these offspring may be being assigned to genotyped males at low confidence when the true father was not genotyped.

Confidence of paternity assignment was also found to decrease when the assigned parents were related, indicating that there may be a bias towards paternity assignment within unrelated mating pairs. However, the effect of parent relatedness of the confidence of paternity is very small (parents with *r* = 0.5 were expected to have a paternity assignment with confidence reduced by 0.04 compared to paternity assignment between parents with *r* = 0) and unlikely to drive a reduction in confidence below our acceptance threshold (0.8; see figure S1.3.2). Indeed, of the offspring with paternity assigned < 0.8 for which we can estimate parent relatedness 45% have a relatedness of zero.

Though it remains possible that the results described in the main text of this article could be driven by a bias in paternity assignment the results presented here suggest that such a bias is unlikely.

**References:**

**Robertson, A. & Hill, W. G., 1984**. Deviations from Hardy-Weinberg proportions: sampling variances and use in estimation of inbreeding coefficients. Genetics 107: 703 (718).

**Weir, B. S. & Cockerham, C. C., 1984**. Estimating F-statistics for the analysis of population structure. Evolution 38: 1358 (1370).

**Benjamini, Y, and Hochberg, Y., 1995**. Controlling the false discovery rate: a practical and powerful approach to multiple testing. Journal of the Royal Statistical Society. Series B (Methodological) 57:289-300.

**Raymond M. & Rousset F, 1995**. GENEPOP (version 1.2): population genetics software for exact tests and ecumenicism. J. Heredity, 86:248-249

**Rousset, F., 2008**. Genepop'007: a complete reimplementation of the Genepop software for Windows and Linux. Mol. Ecol. Resources 8: 103-106.
